# Improving breast cancer risk assessment with neighborhood indicators using a case-control study: insights from All of Us

**DOI:** 10.1016/j.lana.2026.101677

**Published:** 2026-11-02

**Authors:** Sasinya N. Scott, Mary Beth Terry, Tian Gu

**Affiliations:** a Department of Epidemiology, Columbia University Mailman School of Public Health, New York, NY, USA; b SUNY Downstate Health Sciences University, Brooklyn, NY, USA; c Department of Pathology and Laboratory Medicine, Memorial Sloan Kettering Cancer Center, New York, NY, USA; d Department of Medicine, Memorial Sloan Kettering Cancer Center, New York, NY, USA; e Silent Spring Institute, Newton, MA, USA; f Department of Biostatistics, Columbia University Mailman School of Public Health, New York, NY, USA

**Keywords:** Breast cancer, Neighborhood, Race and ethnicity, Risk prediction, Health disparities

## Abstract

**Background:**

Accurate breast cancer risk assessment is needed to reduce disparities. We assessed whether neighborhood indicators could replace race and ethnicity without reducing predictive performance.

**Methods:**

We conducted a matched case–control study utilizing All of Us Research Program data (May 2017–June 2022) among participants assigned female at birth aged ≥18. We created six subgroups: four retrospective, one prospective, and one survey-only. We matched cases with up to three controls, by age (±2 years) and follow-up. We developed three conditional logistic regression models: (1) Model 0 (clinical factors, race, ethnicity), (2) Model 1 (Model 0, neighborhood indicators), and (3) Model 2 (clinical factors, neighborhood indicators). Model performance was assessed using discrimination (AUC), risk stratification (top 20%), and decile-based calibration (logarithmic observed/expected ratios).

**Findings:**

Among 413,457 participants, 46,427 were matched (34,810 controls; 11,617 cases): 29,884 (64.4%) Non-Hispanic (NH) White, 7280 (15.7%) NH Black, and 6308 (13.6%) Hispanic, other groups not reported due to small sample size. Across analytic subgroups, models demonstrated similar but minimal discrimination (pooled AUCs 0.54–0.62), risk stratification (52–234 cases [26.7–37.8%] in the top 20% of risk), and calibration. Replacing race and ethnicity with neighborhood indicators changed AUC by 0.06 and cases in the top 20% of predicted risk by less than 4 percentage points.

**Interpretation:**

Neighborhood indicators may replace race and ethnicity in risk models while preserving performance, despite limited discrimination. Their use may better identify social and structural factors relevant to resource and place-based interventions. Future research should consider race-conscious structural measures to refine prediction.

**Funding:**

10.13039/100000002NIH, 10.13039/100000054NCI, 10.13039/100001006Breast Cancer Research Foundation.

**Research in context**

**Evidence before this study**

To assess the existing evidence on breast cancer risk prediction, from January 1st, 2022 to September 30th, 2026, we searched PubMed, Google Scholar, and EMBASE, Web of Science Core Collection for studies examining breast cancer risk prediction, without restrictions on publication date or language. Our search strategy was ((“Breast Neoplasm” [MeSH] OR “breast cancer”) AND (“Risk Assessment” [MeSH] OR “risk prediction” OR “risk model”) AND (“Health disparities” [MeSH] OR “health disparities” OR “racial disparities”)). We focused on clinically validated risk assessment models, their performance across racial and ethnic populations, and any incorporation of social and neighborhood-level factors in risk prediction. Studies show that many clinically validated models were developed using data from majority White cohorts. In addition, several models use race- and ethnicity-based adjustments within the model and as population-level incidence adjustments rather than estimating risk across racial and ethnic subgroups. Such approaches have been criticized for (1) limited causal justification, (2) limited evaluation across diverse populations, (3) embedding incorrect biological assumptions of race, and (4) potentially obscuring the structural drivers of risk disparities. Consequently, previous studies demonstrate reduced accuracy of these models among minoritized populations compared to White populations, raising concerns about their equity and clinical use.

**Added value of this study**

Using data from the All of Us Research Program, where participant enrollment spanned from May 31, 2017–June 30, 2022, this study contributes to the ongoing discussion about the role of race and ethnicity in breast cancer risk prediction by evaluating neighborhood indicators as more direct measures of structural context and assessing their contribution to risk prediction across racial and ethnic categories. Models incorporating neighborhood indicators demonstrated comparable discrimination, risk stratification, and calibration with models that included race and ethnicity. These findings support continued efforts to determine how neighborhood indicators can best be represented in breast cancer risk models, while also suggesting that removing race and ethnicity from risk models may not compromise predictive performance.

**Implications of all the available evidence**

Our findings indicate that neighborhood-based measures may serve as potential replacements for race and ethnicity within risk prediction. As risk assessment continues to evolve, future research should focus on validating predictive models with explicit measures of structural context that could support more equitable breast cancer risk assessment and reduce disparities.

## Introduction

Population-level breast cancer screening has improved early disease detection and reduced mortality.^1^ However, it often relies on broad age criteria that do not capture the full spectrum of individual risk.^2^ In breast cancer, the mismatch is evident and significant due to substantial disease burden. Breast cancer is the most common cancer among women, with over 300,000 new cases and approximately 42,000 deaths estimated in the United States in 2025.^3^ Screening guidelines focus on women aged 40–74, with those outside the age range screened only if they possess additional risk factors, like family history of breast cancer.^4^ For example, the American Cancer Society recommended in 2007 supplemental annual magnetic resonance imaging (MRI) screening for women with a lifetime risk of 20% or greater.^5^ Recent guideline modifications incorporate a fixed 5-year absolute risk window, as validation studies demonstrate that it provides a more accurate screening threshold than lifetime risk models.^6^ Despite these recommendations, around 4% of invasive cases occur under age 40 and 20% above age 74, leading to 24% of cases falling outside the screening window.^3^ Limitations of using age and family history alone underscore the multidimensional nature of breast cancer and the need for more personalized, risk-based approaches.

Individual risk assessment incorporates multiple predictors to refine screening and prevention strategies, leading to earlier diagnoses even for those outside the standard guidelines.^7^ While risk models are increasingly used, they often perform poorly in populations underrepresented in model development cohorts.^8^ Many early models were based on largely White, racially homogeneous studies that either excluded minoritized individuals, those who have historically experienced marginalization or unequal treatment systematically, such as Black, Hispanic, and Asian people, or lacked statistical power to analyze model performance across diverse groups. Therefore, these models can result in misestimation of risk for racial, ethnic, and socioeconomic minorities, leading to a reinforcement of existing disparities.

Many models include race and ethnicity as convenient substitutes for unmeasured social determinants of health (SDoH). However, doing so without examining the structural context they represent assumes uniform effects across populations and ignores complex interactions.^9^ Race is a social construct that reflects historically and culturally defined fluctuating systems of marginalization and power, not biological traits. Using race uncritically in risk models (i.e., race-adjusted, race-based) may reinforce false notions of biological racial difference and may systematically direct resources away from communities most affected by disparities.^10^ While race remains salient to public health, the field's theoretical and methodological conventions fail to capture the mechanisms through which structural racism influences health.^11^ Therefore, developing alternatives to race-based algorithms is essential for more equitable care.^12^

Structural context represents one pathway through which structural racism operates, influencing the social and built environment that can, in turn, influence health. Neighborhood characteristics such as social cohesion, neighborhood conditions, and physical movement influence health outcomes through physiological effects, changing behavior, and access to health services. Structural racism can exacerbate negative social interaction and environmental conditions that can lead to adverse health. Given structural racism's historical and embedded nature, minoritized communities encounter more adverse structural conditions than White populations, compounding their individual risk and leading to persistent disparities in prevention, diagnosis, and treatment outcomes. Most risk models omit upstream determinants like the social and built environment, despite evidence linking them to risk and disparities.^13^ Neighborhood indicators may capture structural context better than race or ethnicity, potentially improving model validity and fairness. Race-neutral versions fail to explicitly measure the mechanisms through which racism operates, limiting their ability to fully account for systemic drivers compared to race-conscious indicators that quantify dimensions of structural racism and their impact.^14^ For example, measures of residential segregation and racialized economic privilege quantify the degree to which communities are physically or socially isolated, reflecting historical discriminatory policies on neighborhood resource allocation.^15^ Including any form of neighborhood indicator may provide the potential to capture underlying conditions that lead to inequitable risk prediction and disparities, and allow for tailored prevention efforts that address context-based drivers of breast cancer risk.

Given current cancer models' reliance on race and ethnicity and failure to include structural factors, we examined whether incorporating race-neutral neighborhood social and built environment indicators improves breast cancer risk prediction. We hypothesize that these indicators may improve model performance, reduce disparities in predictive accuracy, and offer a more equitable alternative to race-based risk modeling.

## Methods

### Data source

We used data from version 7 of the All of Us (AoU) Research Program national database, controlled tier, where participant enrollment spanned from May 31, 2017 to June 30, 2022.^16^ AoU consolidates participant information from electronic health records (EHR), survey, and zip-code-level indicators.

### Study population

Eligibility included participants 18 years and older who (1) were female in the EHR, and (2) had available AoU consent and demographic information. Participants aged 18 and older were included to evaluate model performance across the full adult AoU population, including younger adults who may develop early onset cancer. Matched analysis consisted of 46,427 women (34,810 controls and 11,617 cases) across six analytic subgroups.

### Study design

To capture temporal relationships, we used a matched case–control design with four retrospective, one prospective, and one survey-only analytic subgroup. The four retrospective groupings included those who were diagnosed with breast cancer (cases) within the EHR before consenting to the AoU study: (1) 0–2 years (Ret-G1), (2) 2–5 years (Ret-G2), (3) 5–10 years (Ret-G3), and (4) more than 10 years (Ret-G4) and their matched controls (Fig. 1). The prospective subgroup included those who were diagnosed with breast cancer within the EHR after AoU consent and their matched controls. Due to the lack of a breast cancer diagnosis date and tumor characteristics (e.g., invasive versus non-invasive status) in the AoU “Personal and Family Health History” (PFHH) survey, participants whose diagnoses were documented solely within this survey were assigned to the survey-only subgroup.

**Fig. 1:**
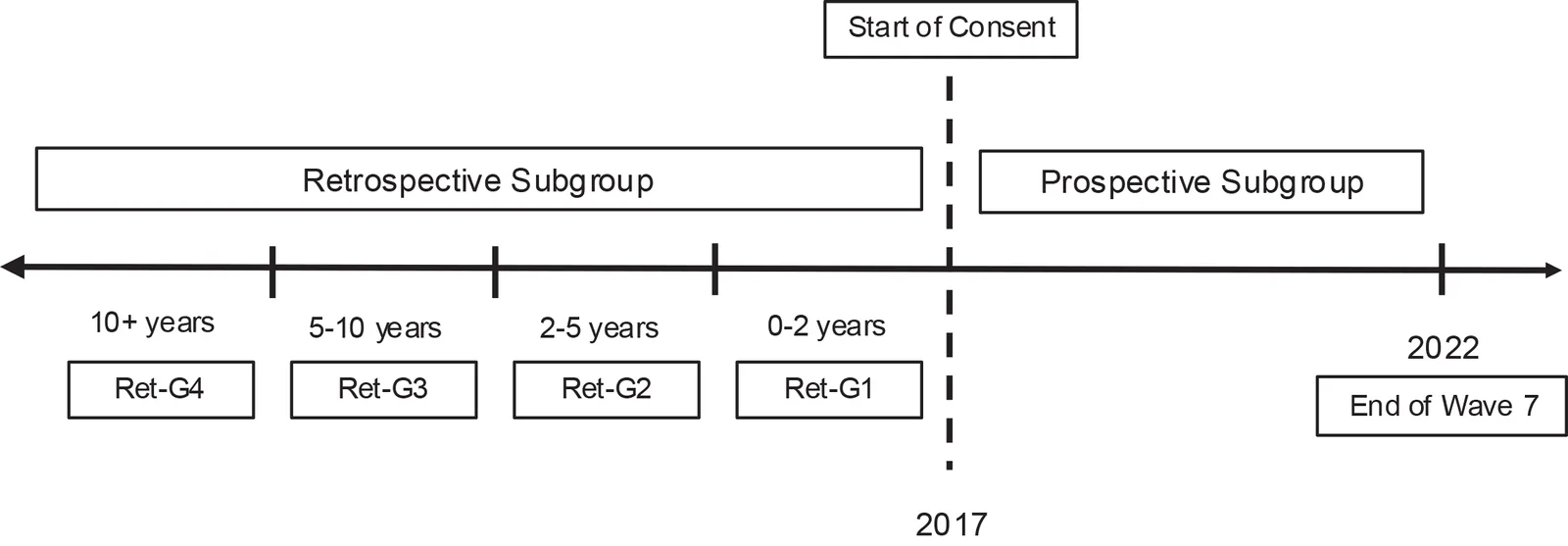
**Analytic subgroups and study timeline.** Ret-G1, retrospective diagnoses 0–2 years before All of Us (AoU) consent with matched controls; Ret-G2, retrospective diagnoses 2–5 years before AoU consent with matched controls; Ret-G3, retrospective diagnoses 5–10 years before AoU consent with matched controls; Ret-G4, retrospective diagnoses 10+ years before AoU consent with matched controls; Prospective, prospective diagnoses after AoU consent with matched controls; and Survey-only, participants with a breast cancer diagnosis determined only by AoU survey with no diagnosis date available.

Participants without a breast cancer diagnosis before or during the study period were included as controls. We used 3:1 matching without replacement based on age (±2 years) and follow-up time that was equal to or exceeded that of the case. Each case could have one to three matches. Cases who remained unmatched were removed from the analytic population.

### Outcome variable

We created a binary outcome variable indicating a breast cancer diagnosis (yes/no) in the EHR and/or PFHH survey (Supplemental Table S1). First, we identified participants in the retrospective and prospective subgroups as having breast cancer if they had a primary malignant neoplasm of female breast diagnosis (i.e., parent code: 137809) within the EHR. Second, for the survey-only subgroup we used self-reported responses to the PFHH survey, classifying participants as having breast cancer. The survey did not distinguish between cancer type. Therefore, the EHR and PFHH survey data sources represent differing case definitions. We addressed the differing case definitions by analyzing them separately and conducting sensitivity analyses. Those without a diagnosis from either source were classified as not having breast cancer.

### Predictor variables

#### Demographic and clinical predictors

Demographic and clinical characteristics were selected based on their established relevance to breast cancer risk and their availability within the AoU database (Supplemental Table S1). Age at consent was calculated as the date of AoU consent minus their date of birth. Race and ethnicity were ascertained from the EHR because these variables are routinely available and consistently coded measures across analytic subgroups, aligning with the study objective of evaluating models built from routinely available data and linked neighborhood measures. Self-reported race and ethnicity were also collected through the "Basics" survey, but analyses used only EHR-recorded race and ethnicity given the substantial percent agreement between the two sources for the major racial (88.5–97.8%) and ethnic categories (96.8%). Although EHR-derived and self-reported measures of race and ethnicity are not conceptually interchangeable, their concordance suggests that the use of EHR-derived race and ethnicity is unlikely to materially alter the primary model comparisons. We combined EHR categories into mutually exclusive race and ethnicity groupings (e.g., Non-Hispanic Black, etc.) for analysis. Due to low numbers and resulting unstable estimates, groups other than Non-Hispanic Black, Hispanic, and Non-Hispanic White were excluded from reporting. Excluded groups included Non-Hispanic Asian, Non-Hispanic Native Hawaiian or Other Pacific Islander, Other (i.e., Middle Eastern and North African [MENA] or multiple race), and Unknown (i.e., skipped, preferred not to say, none indicated, or none of these).

We extracted family history of breast cancer in first-degree relatives from the PFHH survey and previous biopsies and mammograms from the EHR. Previous biopsies were defined as the number of procedures, with same-day procedures counted once. Mammographic history was defined as one mammogram per day per individual. To reduce diagnostic bias, both previous biopsies and mammographic history were only included if they were performed at least one year prior to their breast cancer diagnosis for cases or at least one year before the matched case's breast cancer diagnosis for controls. For the survey-only analytic subgroup, we used their AoU primary consent date as the index date and used any prior biopsies and mammographic history one year prior to that date for cases and their matched controls.

### Neighborhood indicators

To evaluate whether area-based structural measures and residents' lived experiences of neighborhood capture contexts relevant to breast cancer risk, we estimated models using two types of neighborhood indicators: Area Deprivation Index (ADI) linked by ZIP code and three self-reported measures of neighborhood environment. ADI represents average socioeconomic disadvantage calculated using 2017 American Community Survey. The calculation and component measures for ADI are provided in Supplemental Table S2. We used three validated instruments from the SDoH survey, the Social Cohesion Scale (social scale),^17^ the Ross-Mirowsky Disorder Scale (rm scale),^18^ and the Physical Activity Neighborhood Environment Scale (panes scale).^19^ These instruments respectively assessed social connections, values, trust, perceived neighborhood physical and social order, and neighborhood accommodations for physical activity. Survey questions and scoring are detailed in Supplemental Table S3.

### Correlation assessment

To assess the association between race and ethnicity and AoU neighborhood indicators, we calculated Eta-squared ($\eta^{2}$), which quantifies the proportion of variance in each neighborhood indicator explained by differences across race and ethnicity. Low $\eta^{2}$ values indicate that race and ethnicity explain less of the variation in neighborhood indicators, suggesting that these measures capture additional environmental and social information beyond racial and ethnic group membership.

### Sensitivity analyses

First, to examine differences in breast cancer case ascertainment between the EHR (invasive diagnoses) and the PFHH survey (invasive and non-invasive presentations), we replicated our primary analyses using an expanded case definition list that included ductal carcinoma in situ (DCIS). Second, to evaluate whether neighborhood indicator missingness influenced our results, we conducted a complete-case analysis assessing discrimination and risk stratification. Finally, to assess the potential impact of survivorship bias, we conducted a sensitivity analysis comparing participants diagnosed more than 10 years before study consent (Ret-G4) with those diagnosed 0–2 years before study consent (Ret-G1). Among participants in these two retrospective groups, we fit a multivariable logistic regression model with subgroup membership (Ret-G4 versus Ret-G1) as the binary outcome. Predictors included age at consent, number of previous biopsies, number of first-degree relatives with breast cancer, social scale, rm scale, panes scale, ADI, and breast cancer status. The model was fitted separately in each of the 50 multiply imputed datasets, where regression coefficients and standard errors were pooled across imputations using Rubin's Rules. Statistical significance was assessed using pooled Wald tests, including joint Wald tests for predictors modeled using restricted cubic spline (RCS).

### Risk assessment

Three model variations were evaluated (Table 1): (1) Model 0 (Breast Cancer Risk Model [BCRM]): clinical factors, and race and ethnicity, (2) Model 1 (BCRM + neighborhood-based indicators): clinical factors, race and ethnicity, and neighborhood indicators, and (3) Model 2 (Neighborhood-based Risk Model [NBRM]): clinical factors, and neighborhood indicators.

**Table 1:** De novo risk model formulas.

Models	Variables included	Model type
Model 0	breastcancer ∼ T1 + nbiop + nrels + race and ethnicity	BCRM
Model 1	breastcancer ∼ T1 + nbiop + nrels + race and ethnicity + social scale + rm scale + panes scale + ADI	BCRM + neighborhood-based indicators
Model 2	breastcancer ∼ T1 + nbiop + nrels + social scale + rm scale + panes scale + ADI	NBRM

**Abbreviations:** BCRM, Breast Cancer Risk Model (clinical factors, race, ethnicity); NBRM, Neighborhood-Based Risk Model (clinical factors, neighborhood indicators); T1, age at consent; nbiop, number of previous biopsies; nrels, number of first-degree relatives with breast cancer; social scale, Social Cohesion scale; rm scale, Ross-Mirowsky Disorder scale; panes scale, Physical Activity Neighborhood Environment (PANES) scale; ADI, area deprivation index.

Model selection and fit were assessed using Akaike Information Criterion (AIC), Bayesian Information Criterion (BIC), and McFadden's pseudo-R^2^. Optimal model fit was defined by the lowest AIC and BIC values. For pseudo-R^2^, goodness-of-fit values were interpreted as (1) poor (0–0.1), (2) fair and acceptable (0.1–0.2), (3) excellent (0.2–0.4), and (4) suggestive data leakage/overfitting (>0.4). Model performance was evaluated using discrimination, risk stratification, and calibration across analytic subgroups and ethnoracial categories. Discrimination was assessed using area under the curve (AUC), where 0.5 was no better than random chance, 0.5–0.6 equated to minimal discrimination, acceptable was between 0.6–0.7, and good discrimination was 0.7–0.8. To evaluate clinically actionable high-risk strata, we assessed the proportion of breast cancer cases captured within the top 20% of predicted risk for each model. We applied multiple imputation to address missingness in neighborhood indicators, including social scale (n = 30,785; 66.3%), rm scale (n = 30,651; 66.0%), and panes scale (n = 30,776; 66.3%), as detailed in the following section. Given the low ADI missingness (n = 32; 0.1%), analyses including ADI were conducted using complete cases. Potentially nonlinear continuous neighborhood indicators were modeled using five-knot RCS with prespecified knot locations to account for nonlinear associations. For the imputed and complete-case neighborhood models, we estimated conditional predicted probabilities using conditional logistic regression. Within each analytic subgroup, participants were ranked by their predicted risk estimates, and the top 20% of predicted risk was identified. Calibration was evaluated using the logarithm of the observed-to-expected ratio, i.e., log (O/E), which quantifies the direction and magnitude of deviation between observed and expected risk. To account for matching, we fit conditional logistic regression models for all models using the training set within each imputed dataset. Since conditional logistic regression estimates effects within matched sets, only matched strata containing both a case and at least one control contributed to the model. The fitted conditional logistic regression models were applied within each imputed testing dataset to generate individual-level conditional predicted probabilities of breast cancer. Within each imputation, participants were grouped into deciles based on the distribution of conditional predicted probabilities. For each decile, the observed number of breast cancer cases divided by the expected number of breast cancer cases was calculated to determine the observed-to-expected ratios. The observed-to-expected ratios were logarithmically transformed to obtain log (O/E), which symmetrically represents under-estimation and over-estimation around zero, where log (O/E) = 0 indicates perfect calibration with agreement between observed and expected case counts. Decile-specific log (O/E) estimates were then pooled across imputations using Rubin's rules. Log (O/E) indicates both the direction of deviation, where log (O/E) < 0 indicates overestimation (observed < expected) and log (O/E) > 0 indicates underestimation (observed > expected), and its magnitude, where values further from zero indicate greater deviation. Average absolute log (O/E) summarized the magnitude of deviations from an observed-to-expected ratio of one, with lower values indicating closer agreement and zero indicating perfect calibration. All statistical analyses were performed using R (version 4.2 or later) and statistical significance was determined by *p*-value (*p*) < 0.05.

### Ethics

The AoU Research Program protocol was reviewed by the AoU Institutional Review Board (IRB) (Protocol #: 2021-02-TV-001). The study protocol was approved under the AoU Institutional Review Board framework, and all participants provided written informed consent to have their data used for research. All study procedures were conducted in compliance with the AoU's data use agreement. To protect participant confidentiality, participant data were de-identified and cell sizes less than 20 individuals were censored.

### Statistical analysis

Our primary goal was to evaluate whether risk models incorporating neighborhood indicators performed as well or better than models that incorporated race and ethnicity. Descriptive statistics were generated separately for each analytic subgroup. To assess the functional form of each continuous predictor, we compared conditional logistic regression models with linear and five-knot RCS terms using a likelihood ratio test and AIC.^20^ Lower AIC values supported the selection of the RCS model for ADI, social scale, age at consent, and panes scale, while the linear model was retained for the rm scale. Normality assumptions for continuous variables were assessed and due to non-normal distributions, they were descriptively compared using Wilcoxon rank-sum tests. Categorical variables were compared using Fisher's exact test due to expected small cell counts.

For neighborhood indicators with >10% missingness, we assessed the missing completely at random (MCAR) assumption using conditional logistic regression by modeling a binary missingness indicator (1 = missing and 0 = observed) as a function of observed covariates. Significant associations (*p* < 0.05) were interpreted as evidence against the MCAR assumption, indicating that missingness may be dependent on observed or unobserved factors. Since distinguishing between missing at random (MAR) and missing not at random (MNAR) is not possible using observed data alone, we proceeded under a MAR framework for handling missing data, while acknowledging that the MAR assumption cannot be empirically verified. All predictive modeling was performed using an 80/20 random split at the matched-set level, stratified by subgroup, with 80% of the matched sets allocated for training and the remaining 20% for testing. Model coefficients estimated in the training dataset were held fixed and applied without re-estimation in the testing dataset, to promote independent validation of model performance. Multiple imputation was performed after the training-testing split, with imputation models estimated using the training data to minimize information leakage into the testing set. We imputed missing data in the neighborhood-based indicators, including social scale (n = 30,785; 66.3%), rm scale (n = 30,651; 66.0%), and panes scale (n = 30,776; 66.3%), within our analytic population. ADI was not imputed due to lower than 10% missingness (n = 32; 0.1%). Imputation was performed using predictive mean matching using multiple imputation via chained equations (MICE). The imputation model included age at consent, race, ethnicity, number of previous biopsies, number of previous mammograms, number of first-degree relatives with breast cancer, and breast cancer outcome status. The outcome variable was included in the imputation to maintain the associations between the predictors and outcome. To ensure the stability of our estimates, we conducted a sensitivity analysis comparing different m values of imputations [m = 10–60, units of 10] using the mean pooled AUC change and mean between variance change to determine which imputation m value had the first evidence of consistent stability and where additional imputations did not further improve stability. We determined that m = 50 gave the best balance of statistical stability and computational feasibility and used that in our final analyses. Final estimates were pooled across imputations using Rubin's Rules.^21^

### Role of the funding source

Our funders had no role in the study design, data collection, data analysis, interpretation or writing of this report.

## Results

Table 2 summarizes participant characteristics across analytic subgroups. Race and ethnicity distributions differed between cases and controls in most analytic subgroups (*p* < 0.05), except Ret-G3 (*p* = 0.055). Among the neighborhood-based indicators, social scale was similar in retrospective subgroups (all *p* > 0.05) but differed in the prospective and survey-only groups (*p* < 0.05 for both). ADI was lower among cases in half the subgroups, including Ret-G3, Ret-G4, and survey-only (all *p* < 0.001), while panes scale was higher among cases in Ret-G1, Ret-G3, and survey-only (*p* = 0.004, 0.032, and 0.010, respectively).

**Table 2:** Demographic, clinical, and neighborhood-based indicators of study participants by analytic subgroup, N = 46,427.

	Prospective			Ret-G1			Ret-G2			Ret-G3			Ret-G4			Survey-Only		
	Case	Control	*p*-value^a^	Case	Control	*p*-value^a^	Case	Control	*p*-value^a^	Case	Control	*p*-value^a^	Case	Control	*p*-value^a^	Case	Control	*p*-value^a^
**Total**	3081 (100%)	9231 (100%)		1309 (100%)	3918 (100%)		1679 (100%)	5036 (100%)		1516 (100%)	4541 (100%)		934 (100%)	2794 (100%)		3098 (100%)	9290 (100%)	
**Age at study consent (mean (SD))**	60 (13)	60 (13)	0.72	62 (12)	62 (12)	0.59	64 (10)	64 (10)	0.69	67 (10)	67 (10)	0.49	70 (9)	69 (9)	0.44	64 (11)	64 (11)	0.48
**Race and ethnicity (N (%))**																		
Non-Hispanic Black	410 (13.3%)	1741 (18.9%)	<0.001	148 (11.3%)	690 (17.6%)	<0.001	219 (13.0%)	808 (16.0%)	<0.001	223 (14.7%)	753 (16.6%)	0.055	89 (9.5%)	407 (14.6%)	0.004	217 (7.0%)	1575 (17.0%)	<0.001
Hispanic	444 (14.4%)	1416 (15.3%)		183 (14.0%)	565 (14.4%)		198 (11.8%)	732 (14.5%)		170 (11.2%)	604 (13.3%)		117 (12.5%)	332 (11.9%)		198 (6.4%)	1349 (14.5%)	
Non-Hispanic White	2007 (65.1%)	5484 (59.4%)		865 (66.1%)	2387 (60.9%)		1165 (69.4%)	3193 (63.4%)		1028 (67.8%)	2934 (64.6%)		679 (72.7%)	1906 (68.2%)		2454 (79.2%)	5782 (62.2%)	
**Number of Previous Biopsies (mean (SD))**	0.1 (0.3)	0.02 (0.2)	<0.001	0.1 (0.3)	0.02 (0.2)	<0.001	0.05 (0.3)	0.01 (0.1)	<0.001	0.05 (0.2)	0.01 (0.1)	<0.001	0.04 (0.3)	0.00 (0.1)	<0.001	0.05 (0.3)	0.03 (0.2)	<0.001
**Number of first-degree relatives with breast cancer (mean (SD))**	0.1 (0.4)	0.1 (0.3)	<0.001	0.2 (0.4)	0.1 (0.3)	<0.001	0.2 (0.4)	0.1 (0.3)	<0.001	0.2 (0.5)	0.1 (0.3)	<0.001	0.2 (0.5)	0.1 (0.3)	<0.001	0.3 (0.5)	0.1 (0.3)	<0.001
**Number of previous mammograms (mean (SD))**	0.4 (1.4)	0.2 (1.1)	<0.001	0.4 (1.2)	0.2 (0.9)	<0.001	0.2 (1.0)	0.1 (0.6)	<0.001	0.2 (0.8)	0.1 (0.5)	<0.001	0.1 (0.6)	0.01 (0.2)	<0.001	0.1 (0.7)	0.2 (1.2)	<0.001
**Neighborhood-based indicators**																		
**Social cohesion scale (mean (SD))**	15.5 (3.0)	15.1 (3.2)	0.022	15.2 (3.1)	15.3 (3.1)	0.56	15.3 (3.2)	15.1 (3.1)	0.23	15.4 (3.1)	15.5 (3.1)	0.34	15.7 (3.0)	15.4 (3.2)	0.13	15.5 (3.1)	15.2 (3.1)	0.017
Unknown (Missing) (N (%))	1900 (61.7%)	6676 (72.3%)		836 (63.9%)	2758 (70.4%)		1017 (60.6%)	3533 (70.2%)		908 (59.9%)	3175 (69.9%)		552 (59.1%)	1905 (68.2%)		1073 (34.6%)	6452 (69.5%)	
**Ross-Mirowsky disorder scale (mean (SD))**	19.5 (6.2)	19.9 (6.5)	0.10	19.8 (6.8)	19.7 (6.3)	0.93	19.1 (6.1)	19.6 (6.2)	0.072	19.2 (5.9)	19.5 (6.1)	0.67	18.9 (6.2)	18.9 (5.9)	0.73	19.2 (6.1)	19.6 (6.4)	0.082
Unknown (Missing) (N (%))	1889 (61.3%)	6660 (72.1%)		833 (63.6%)	2748 (70.1%)		1011 (60.2%)	3520 (69.9%)		900 (59.4%)	3163 (69.7%)		549 (58.8%)	1895 (67.8%)		1057 (34.1%)	6426 (69.2%)	
**Physical activity neighborhood environment scale (mean (SD))**	13.8 (6.5)	13.4 (6.2)	0.12	14.4 (6.3)	13.4 (6.3)	0.004	13.5 (6.1)	13.5 (6.3)	0.94	14 (6.1)	13.3 (6.2)	0.032	13.7 (6.3)	13.1 (6.3)	0.15	14 (6.3)	13.4 (6.2)	0.010
Unknown (Missing) (N (%))	1894 (61.4%)	6685 (72.4%)		833 (63.6%)	2761 (70.5%)		1016 (60.5%)	3532 (70.1%)		902 (59.5%)	3179 (70.0%)		552 (59.1%)	1901 (68.0%)		1068 (34.5%)	6453 (69.5%)	
**Area deprivation index (mean (SD))**	0.32 (0.1)	0.32 (0.1)	0.25	0.32 (0.1)	0.32 (0.1)	0.13	0.32 (0.1)	0.32 (0.1)	0.071	0.31 (0.1)	0.32 (0.1)	<0.001	0.29 (0.1)	0.32 (0.1)	<0.001	0.31 (0.1)	0.32 (0.1)	<0.001
Unknown (Missing) (N (%))	<20 (−)	<20 (−)		<20 (−)	<20 (−)		<20 (−)	<20 (−)		<20 (−)	<20 (−)		<20 (−)	<20 (−)		<20 (−)	<20 (−)	

**Abbreviations:** SD, standard deviation. Prospective, prospective diagnoses after All of Us (AoU) consent with matched controls; Ret-G1, retrospective diagnoses 0–2 years before AoU consent with matched controls; Ret-G2, retrospective diagnoses 2–5 years before AoU consent with matched controls; Ret-G3, retrospective diagnoses 5–10 years before AoU consent with matched controls; Ret-G4, retrospective diagnoses 10+ years before AoU consent with matched controls; and Survey-only, participants with a breast cancer diagnosis determined only by AoU survey with no diagnosis date available.

Race and ethnicity categories not shown in the table were as follows (case/control (count(%))): Non-Hispanic Asian: Prospective: 72 (2.3%)/221 (2.4%), Ret-G1: 46 (3.5%)/98 (2.5%), Ret-G2: 30 (1.8%)/73 (1.4%), Ret-G3: 30 (2.0%)/73 (1.6%), Ret-G4: <20 (−)/52 (1.9%), and Survey-Only: 82 (2.6%)/186 (2.0%); Non-Hispanic Native Hawaiian or Other Pacific Islander: <20 (−) for cases and controls in Ret-G1, Ret-G3, Ret-G4, and Survey-only, Ret-G2: <20 (−)/34 (0.6%); Other/Unknown: Prospective: 145 (4.7%)/360 (3.9%), Ret-G1: 66 (5.0%)/172 (4.4%), Ret-G2: 65 (3.9%)/196 (3.9%), Ret-G3: 65 (4.3%)/173 (3.8%), Ret-G4: 34 (3.6%)/95 (3.4%) and Survey-Only: 144 (4.6%)/393 (4.2%). Participants in these categories were included in the mean (SD) and *p*-values of all other descriptive statistics presented in the table.

a Continuous variables are reported as mean (SD) and compared using Wilcoxon rank-sum tests; Categorical variables are reported as n (%) and compared using Fisher's exact tests. Statistical significance was determined by *p*-value (*p*) < 0.05.

### Model performance

When comparing our three de novo models, (1) Model 0: BCRM, (2) Model 1: BCRM + neighborhood indicators, and (3) Model 2: NBRM, we determined that Model 1 had the lowest AIC, and highest pseudo R^2^, while Model 0 had the lowest BIC (Supplemental Table S4). Model 2, which excluded race and ethnicity, performed similarly to Models 0 and 1.

### Correlation

We assessed how much each neighborhood indicator variance could be attributed to race and ethnicity across all subgroups (Supplemental Table S5). We found that race and ethnicity accounted for 12% of the variance in ADI, indicating that knowing the racial and ethnic background of participants can provide some predictive value for area deprivation. Conversely, race and ethnicity accounted for little perceived neighborhood indicator variance, accounting for only 0.1%, 3.0%, and 4.0% in panes scale, social scale, and rm scale, respectively.

### Sensitivity analyses

We conducted a sensitivity analysis to determine concordance between our EHR-based diagnoses and the survey-based indications of breast cancer (Supplemental Table S6). Among 6964 participants who self-reported a history of breast cancer in the survey, 58.0% (n = 4042) also were identified as having invasive or DCIS in the EHR. Of 8909 participants who had EHR confirmed invasive or DCIS, 45.4% (n = 4042) also had a self-reported history of breast cancer. The expanded case definition increased the number of cases by 232 participants in the prospective subgroup, 132 in Ret-G1, 21 in Ret-G2, and <20 in Ret-G3 and Ret-G4. The corresponding control counts also increased because controls were rematched to the expanded set of cases. The survey-only subgroup decreased by 176 cases because participants who had previously been classified as survey-only were reclassified as EHR-confirmed cases after DCIS diagnoses were incorporated into the EHR definition.

We also repeated the discrimination analyses using the expanded invasive-plus-DCIS case definition (Supplemental Table S7). Overall AUC estimates were similar to those obtained using the invasive-only definition, although the magnitude and direction of differences varied across analytic subgroups and racial and ethnic categories. Across the three models, AUC differences between the invasive-only and invasive-plus-DCIS analyses were small. These findings suggest that inclusion of DCIS changed case classification and increased overlap between EHR and survey ascertainment but did not materially alter the overall pattern of model discrimination.

To evaluate whether the missingness observed for the neighborhood indicators influenced the results, we conducted a complete-case analysis (Supplemental Table S8). We found that across the analytic subgroups, overall AUCs were similar to those from the multiple-imputation analyses, although several race- and ethnicity-specific subgroup estimates were more variable because of the substantially reduced sample sizes. These findings suggest that the overall comparative performance of Models 0–2 was generally consistent between the multiple-imputation and complete-case analyses, although subgroup-specific estimates should be interpreted cautiously because of the extensive missing data.

In our sensitivity analysis evaluating potential survivorship bias, we found evidence that Ret-G4 participants had lower odds of having one previous biopsy than participants in Ret-G1 (adjusted OR = 0.32, 95% CI: 0.13–0.79, *p* = 0.013) and differed with respect to age at AoU consent (overall Wald χ^2^ = 130.0, *p* < 0.001). No evidence of differences was observed for the remaining variables. These findings indicate limited differences in the measured characteristics between Ret-G4 and Ret-G1, although survivorship bias cannot be ruled out.

### Discrimination

Across analytic subgroups, discrimination was similar across the three models, with the pooled AUCs ranging from 0.54 to 0.62 (Fig. 2a and b). Models 1 and 2 demonstrated higher discrimination than Model 0 in the Ret-G4 subgroup (AUCs approximately 0.60 versus 0.54), while discrimination was otherwise comparable across the three models. AUCs among Hispanic participants were lower (0.41–0.65), compared to Non-Hispanic White participants (0.51–0.60) and Non-Hispanic Black participants (0.48–0.62), though all values indicated minimal discrimination. Within each racial and ethnic category, discrimination was similar across the three models with the exception of subgroup Ret-G4 where Models 1 and 2 had higher AUCs than Model 0.

**Fig. 2:**
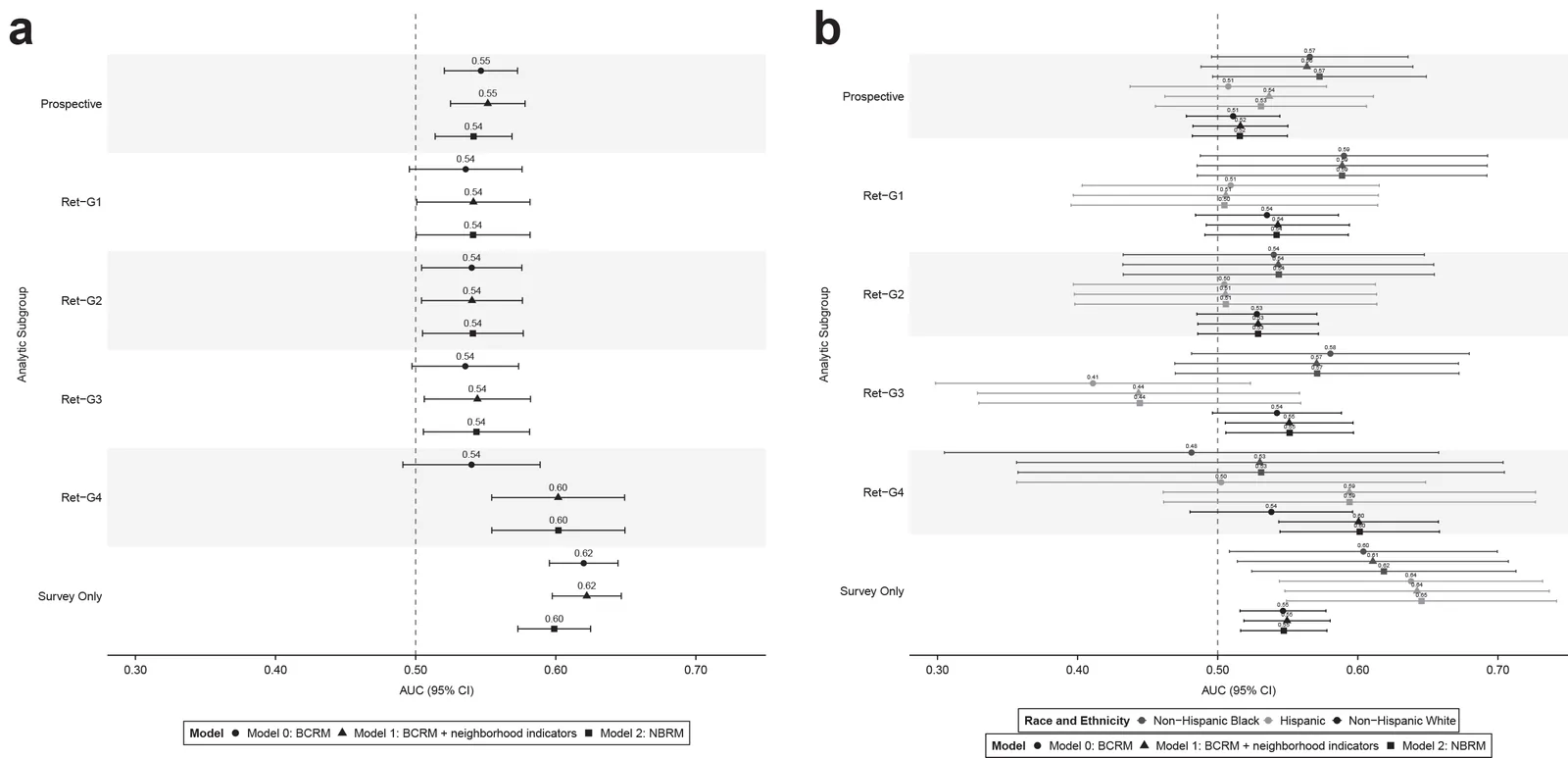
**Model discrimination (AUC) for models 0, 1, and 2 by analytic subgroup, race, and ethnicity.** Model Discrimination, measured using area under the curve (AUC) for models 0 (BCRM), 1 (BCRM + neighborhood indicators), and 2 (NBRM). (a) AUC by analytic subgroup. (b) AUC by analytic subgroup, race, and ethnicity. Points represent AUC estimates, and error bars represent 95% confidence intervals. The dashed line at AUC = 0.50 represents no discrimination. **Abbreviations:** BCRM, Breast Cancer Risk Model (clinical factors, race, ethnicity); NBRM, Neighborhood-Based Risk Model (clinical factors, neighborhood indicators); Prospective, prospective diagnoses after All of Us (AoU) consent with matched controls; Ret-G1, retrospective diagnoses 0–2 years before AoU consent with matched controls; Ret-G2, retrospective diagnoses 2–5 years before AoU consent with matched controls; Ret-G3, retrospective diagnoses 5–10 years before AoU consent with matched controls; Ret-G4, retrospective diagnoses 10+ years before AoU consent with matched controls; and Survey-only, participants with a breast cancer diagnosis determined only by AoU survey with no diagnosis date available.

### Risk stratification

To evaluate our models' ability to identify individuals within a clinically actionable high-risk stratum, we assessed the proportion of breast cancer cases captured in the top 20% of predicted risk (≥80th percentile) (Table 3). We determined across analytic subgroups and models, the proportion of cases captured within the top 20% of predicted risk ranged from 52 to 234 cases (26.7–37.8%). Models 1 and 2 had similar proportion of cases within the top 20% of predicted risk (55–234 cases; 27.6–37.8%) compared to Model 0 (52–220 cases; 26.7–35.5%) across most analytic subgroups, with absolute difference less than four percentage points. Although estimates varied in magnitude, complete case sensitivity analyses, conducted using the complete-case analytic sample rather than the 20% testing sample used in the primary multiply imputed analyses, indicated that risk stratification was consistent across subgroups (81–430 cases; 28.9–33.2%).

**Table 3:** Proportion of breast cancer cases captured in the top 20% of predicted risk across models.

Subgroup	Cases captured in the top 20% of predicted risk, n/N (%) [95% CI]		
	Model 0^a^	Model 1^b^	Model 2^c^
Prospective	165/617 (26.7%) [23.2%–30.2%]	179/616 (29.1%) [25.3%–32.9%]	170/616 (27.6%) [23.6%–31.5%]
Ret-G1	84/262 (32.1%) [26.4%–37.7%]	86/262 (32.8%) [26.8%–38.9%]	87/262 (33.2%) [27.2%–39.5%]
Ret-G2	101/336 (30.1%) [25.2%–35.0%]	107/336 (31.9%) [26.6%–37.3%]	101/336 (30.1%) [24.6%–35.4%]
Ret-G3	88/304 (28.9%) [23.8%–34.0%]	99/303 (32.7%) [27.0%–38.1%]	95/303 (31.4%) [25.7%–37.1%]
Ret-G4	52/187 (27.8%) [21.4%–34.2%]	55/187 (29.4%) [22.6%–36.4%]	56/187 (29.9%) [22.8%–36.5%]
Survey-Only	220/620 (35.5%) [31.7%–39.3%]	234/620 (37.8%) [33.7%–41.8%]	227/620 (36.6%) [32.5%–40.7%]

Counts represent rounded means across imputation. Percentages and 95% confidence intervals (CI) were pooled across imputations. Small differences in denominators across models reflect model-specific exclusions and retention of matched case-control sets. **Abbreviations:** BCRM, Breast Cancer Risk Model (clinical factors, race, ethnicity); NBRM, Neighborhood-Based Risk Model (clinical factors, neighborhood indicators); T1, age at consent; nbiop, number of previous biopsies; nrels, number of first-degree relatives with breast cancer; social scale, Social Cohesion scale; rm scale, Ross-Mirowsky Disorder scale; panes scale, Physical Activity Neighborhood Environment (PANES) scale; ADI, area deprivation index; Prospective, prospective diagnoses after All of Us (AoU) consent with matched controls; Ret-G1, retrospective diagnoses 0–2 years before AoU consent with matched controls; Ret-G2, retrospective diagnoses 2–5 years before AoU consent with matched controls; Ret-G3, retrospective diagnoses 5–10 years before AoU consent with matched controls; Ret-G4, retrospective diagnoses 10+ years before AoU consent with matched controls; and Survey-only, participants with a breast cancer diagnosis determined only by AoU survey with no diagnosis date available.

a Model 0 (BCRM): breastcancer ∼ T1 + nbiop + nrels + race and ethnicity.

b Model 1 (BCRM + neighborhood indicators): breastcancer ∼ T1 + nbiop + nrels + race and ethnicity + social scale + rm scale + panes scale + ADI.

c Model 2 (NBRM): breastcancer ∼ T1 + nbiop + nrels + social scale + rm scale + panes scale + ADI.

### Calibration

Overall, calibration patterns were similar across the three models, with log (O/E) values varying across analytic subgroups and racial and ethnic categories (Fig. 3a–c). Model 1 demonstrated the lowest average absolute log (O/E) in most comparisons, with some variation across subgroups (Table 4). Non-Hispanic White participants' values were generally centered closer to zero compared to Non-Hispanic Black and Hispanic participants, though patterns were not universal for all subgroup–model combinations. Overall, since all three models were evaluated within a case–control sample rather than the source population, these analyses should be interpreted as relative assessments of calibration patterns rather than formal evaluations of population-level absolute risk calibration.

**Fig. 3:**
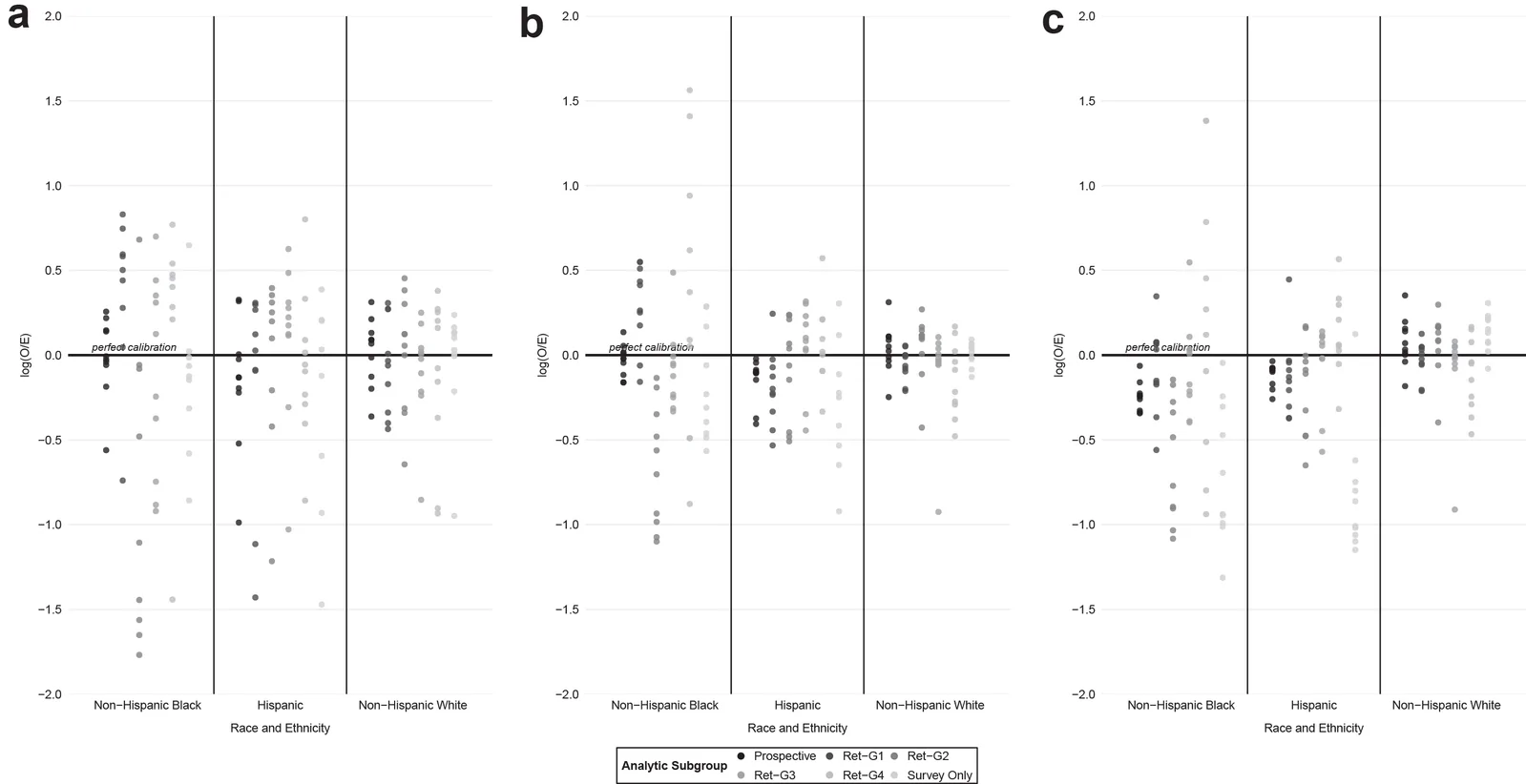
**Calibration across deciles by analytic subgroup, race, and ethnicity.** Calibration using logarithmic observed-to-expected ratio (log (O/E)) across deciles by analytic subgroup, race, and ethnicity. (a) Calibration for Model 0 (BCRM). (b) Calibration for Model 1 (BCRM + neighborhood indicators). (c) Calibration for Model 2 (NBRM). **Abbreviations:** BCRM, Breast Cancer Risk Model (clinical factors, race, ethnicity); NBRM, Neighborhood-Based Risk Model (clinical factors, neighborhood indicators); log (O/E), logarithmic observed−to−expected ratio, where values closer to 0 indicate better calibration; Prospective, prospective diagnoses after All of Us (AoU) consent with matched controls; Ret-G1, retrospective diagnoses 0–2 years before AoU consent with matched controls; Ret-G2, retrospective diagnoses 2–5 years before AoU consent with matched controls; Ret-G3, retrospective diagnoses 5–10 years before AoU consent with matched controls; Ret-G4, retrospective diagnoses 10+ years before AoU consent with matched controls; and Survey-only, participants with a breast cancer diagnosis determined only by AoU survey with no diagnosis date available. Other racial and ethnic groups were not presented because low numbers within deciles resulted in unstable estimates and missingness across deciles.

**Table 4:** Average absolute logarithmic observed-to-expected ratio across deciles for each model by subgroup and race and ethnicity.

Analytic subgroup	Race and ethnicity	Average absolute log (O/E)		
		Model 0^a^	Model 1^b^	Model 2^c^
Prospective	Non-Hispanic Black	0.16	0.07	0.24
	Hispanic	0.29	0.15	0.12
	Non-Hispanic White	0.16	0.10	0.12
Ret-G1	Non-Hispanic Black	0.53	0.34	0.20
	Hispanic	0.42	0.24	0.21
	Non-Hispanic White	0.23	0.08	0.08
Ret-G2	Non-Hispanic Black	0.98	0.65	0.61
	Hispanic	0.38	0.24	0.25
	Non-Hispanic White	0.27	0.16	0.16
Ret-G3	Non-Hispanic Black	0.51	0.19	0.21
	Hispanic	0.37	0.21	0.17
	Non-Hispanic White	0.19	0.13	0.13
Ret-G4	Non-Hispanic Black	0.57	0.69	0.67
	Hispanic	0.32	0.18	0.21
	Non-Hispanic White	0.37	0.21	0.20
Survey-Only	Non-Hispanic Black	0.31	0.32	0.70
	Hispanic	0.49	0.39	0.85
	Non-Hispanic White	0.20	0.06	0.15

**Abbreviations:** BCRM, Breast Cancer Risk Model (clinical factors, race, ethnicity); NBRM, Neighborhood-Based Risk Model (clinical factors, neighborhood indicators); T1, age at consent; nbiop, number of previous biopsies; nrels, number of first-degree relatives with breast cancer; social scale, Social Cohesion scale; rm scale, Ross-Mirowsky Disorder scale; panes scale, Physical Activity Neighborhood Environment (PANES) scale; ADI, area deprivation index; Prospective, prospective diagnoses after All of Us (AoU) consent with matched controls; Ret-G1, retrospective diagnoses 0–2 years before AoU consent with matched controls; Ret-G2, retrospective diagnoses 2–5 years before AoU consent with matched controls; Ret-G3, retrospective diagnoses 5–10 years before AoU consent with matched controls; Ret-G4, retrospective diagnoses 10+ years before AoU consent with matched controls; and Survey-only, participants with a breast cancer diagnosis determined only by AoU survey with no diagnosis date available.

a Model 0 (BCRM): breastcancer ∼ T1 + nbiop + nrels + race and ethnicity.

b Model 1 (BCRM + neighborhood indicators): breastcancer ∼ T1 + nbiop + nrels + race and ethnicity + social scale + rm scale + panes scale + ADI.

c Model 2 (NBRM): breastcancer ∼ T1 + nbiop + nrels + social scale + rm scale + panes scale + ADI.

## Discussion

Breast cancer risk prediction models incorporating neighborhood indicators demonstrated comparable performance to models that incorporated race and ethnicity, though minimal, with a slightly larger improvement observed for Ret-G4. Neighborhood-based models identified a similar proportion of breast cancer cases within the highest predicted risk group as models incorporating race and ethnicity. Overall, the comparable performance of neighborhood-based models suggests that neighborhood indicators may provide an alternative approach by incorporating contextual information into breast cancer risk prediction.

Although neighborhood-based models performed comparably to models incorporating race and ethnicity, their utility depends on how neighborhood context is conceptualized and measured. The neighborhood indicators evaluated in this study were race-neutral in design, in that they did not explicitly incorporate race and ethnicity into their items or scoring (i.e., race-based) or capture environmental features explicitly accounting for the racialized policies and histories that shaped them (i.e., race-conscious).^22^ While the neighborhood questionnaires used in AoU were drawn from established instruments, documentation of their development reveals little detail on the demographic composition of the populations used in cognitive testing. Given the lack of racial and ethnic diversity in studies at the time these tools were developed, protective features of minoritized communities may have been overlooked or misclassified.^23^ For example, by focusing on a narrow definition of “order” in the Ross-Mirowsky Disorder Scale, it failed to recognize that protective features like active street life or graffiti can represent social cohesion, collective efficacy, and cultural reclamation.^24^, ^25^ These considerations raise the possibility that the instruments may capture neighborhood experiences differently across racial and ethnic groups, although this was not directly evaluated in our study.

Prior work by Sampson and Raudenbush,^26^ who critiqued the “broken window” theory for embedding racialized perceptions of disorder into neighborhood assessments, found that greater concentrations of Black people were associated with higher perceptions of disorder, with the strongest effect observed for White residents, even after accounting for observed disorder and socioeconomic factors, suggesting that widely used perceptions-based scales may reflect not just environmental features but also racialized interpretations of place.^27^ Similarly, Riley noted that commonly used neighborhood measures often fail to capture historic and spatial variations in community development and the built environment.^28^ They insisted that historic context could be better accessed by incorporating more meaningful geographic units, sources, and challenging normative ideas of race.

While the neighborhood indicators were race-neutral in design, they should not be interpreted as independent of the structural context that has shaped neighborhood environments and the residents' experiences within them. Rather, the neighborhood perceptions they capture reflect broad racialized processes.^15^ Nonetheless, these indicators shift the focus from using race and ethnicity as proxies for structural disadvantage toward neighborhood characteristics that may reflect potentially modifiable social and environmental conditions relevant to disparities. Future work should evaluate whether integration of race-conscious structural measures provides value for breast cancer risk prediction. In addition, future studies should examine perceptions of neighborhoods across racial and ethnic groups within smaller geographic areas. Local norms and community histories may shape perceptions not fully captured by national survey instruments. Exploring these dynamics could help determine whether the scales include inherent biases or whether the responses are shaped by experiences within geographic units.

Model performance was consistent across analytic subgroups, although improvement was greater for Ret-G4. Ret-G4 represents long-term breast cancer survivors, as participants in this subgroup survived and enrolled in AoU more than 10 years after diagnosis. Individuals in Ret-G4 may differ from other subgroups by neighborhood-related characteristics, which may contribute to greater predictive contribution of neighborhood indicators. However, neighborhood characteristics were assessed more than 10 years after diagnosis and may not reflect the neighborhood conditions experienced at the time of breast cancer development. Therefore, the observed difference in Ret-G4 may reflect survivor and selection effects, the timing of neighborhood assessment, or the smaller sample size rather than a true improvement in model discrimination attributable to the neighborhood indicators. Thus, these findings should be interpreted with caution.

### Limitations

First, our analytic sample relied on breast cancer case ascertainment from both the EHR-confirmed and the self-reported PFHH survey. Concordance between EHR- and survey-based breast cancer ascertainment was incomplete. Our sensitivity analysis showed that expanding the EHR case definition to include DCIS resulted in some participants being reclassified from the survey-only to the EHR-confirmed subgroups but not all. Thus, this suggests that differences in breast cancer diagnosis case definition explained only a small proportion of discordance.

The remaining discordance may reflect differences in the scope and timing of information captured by the two data sources. Although AoU has over 430 enrollment sites currently across the U.S., they are limited to collecting data from their participating network. AoU EHR may not capture clinical encounters that occur outside their participating network. Consequently, case ascertainment from the EHR has the potential to underestimate the true disease prevalence due to missing data from non-AoU-participating healthcare providers. Neither ascertainment approach (survey or EHR) completely captures breast cancer history. While our primary time-defined analyses (i.e., four retrospective subgroups and the prospective subgroup) were not driven by survey-only case definitions, we cannot exclude the possibility that some breast cancer diagnoses were incompletely captured within the available EHR records. Moreover, incomplete EHR case ascertainment could have influenced estimates of model performance, especially if EHR completeness varied systematically by participant characteristics, potentially biasing prediction model results.

Another limitation is that we observed a high rate of missingness for the SDoH survey, due to the lag of the survey distribution timeline and response rate. The SDoH survey was implemented in late 2021, which was after a large proportion of the version 7 AoU cohort had been enrolled beginning in 2017.^29^ Since this survey was a late addition to the enrollment protocol, many participants had not yet reached that module by the data cutoff. For retrospective cases, neighborhood characteristics were measured after breast cancer diagnosis and may not reflect the neighborhood context during the relevant exposure period. Although multiple imputation was used to address substantial missingness under the MAR assumption, it does not resolve the temporal limitations of retrospectively measured neighborhood characteristics. We therefore analyzed the prospective subgroup separately and interpret the retrospective findings cautiously.

We observed comparable predictive performance between models that included measures of neighborhood context and those that included race and ethnicity. Our findings suggest that neighborhood context may offer an alternative to the use of race and ethnicity within breast cancer risk prediction models. Future research should evaluate direct race-conscious structural measures and survey instruments that better capture the neighborhood experiences of diverse populations to assess their potential use in breast cancer risk prediction.

## Contributors

SS—conceived the original idea and developed the study design and methodology with support from MBT.

SS—developed the analysis plan, conducted the data analysis, and interpreted results with input from MBT and TG.

SS, MBT, TG—had access to the data used in this study through the All of Us Researcher Workbench and verified the underlying data.

SS—wrote the manuscript with support from MBT and TG.

MBT and TG—provided oversight and guidance throughout the study.

All authors have read and approved the final version of the manuscript and supporting documents.

## Data sharing statement

The version 7 controlled tier data are available through the All of Us Research Program Database.

## Declaration of interests

The authors declare no competing interests.
