# Supplementary material for: Improving breast cancer risk assessment with neighborhood indicators using a case-control study: insights from All of Us
Source: Lancet Reg Health Am. 2026 Nov 2;66:101677. doi: 10.1016/j.lana.2026.101677 (PMC13755468; doi:10.1016/j.lana.2026.101677)
Supplement: Supplementary file 1 [file mmc1.pdf]

Improving Breast Cancer Risk Assessment with Neighborhood Indicators Using a Case-Control Study: Insights from All of Us

Table of Contents

Supplemental Table 1. Predictor and Outcome Variables Available in All of Us (AoU) Research Data. 1

Supplemental Table 2. American Community Survey (ACS) 2017 Measures for Area Deprivation Index Calculation[a]. 6

Supplemental Table 3. Neighborhood-Based Indicators. 6

Supplemental Table 4. DeNovo Model Fit Across Analytic Subgroups. 9

Supplemental Table 5. Neighborhood-Based Indicator Variance Explained by Race and Ethnicity. 10

Supplemental Table 6. Analytic Subgroup Population by Invasive and Invasive + DCIS Datasets. 10

Supplemental Table 7. Analytic Subgroup Discrimination Comparison Between Invasive and Invasive + DCIS Datasets Across Models by Race and Ethnicity. 10

Supplemental Table 8. Analytic Subgroup Discrimination Comparison Between Imputed and Complete Case Datasets Across Models by Race and Ethnicity. .. 12

Supplemental Table 1. Predictor and Outcome Variables Available in All of Us (AoU) Research Data.

Measure	Concept Id[b]	Source/Dataset
**Unique patient identifier**	–	Survey/Person
**Age at consent[a]**	–	Survey/Person
**Sex assigned at birth**		
Female	8532	EHR/Person
**Race and ethnicity**		
Non-Hispanic Asian	Not Hispanic or Latino (38003564)/Asian (8515)	EHR/ Person
Non-Hispanic American Indian or Alaska Native	Not Hispanic or Latino (38003564)/American Indian or Alaska Native (8657)	EHR/ Person
Non-Hispanic Black	Not Hispanic or Latino (38003564)/Black (8516)	EHR/ Person
Hispanic	Hispanic or Latino (38003563), includes no other race code.	EHR/ Person
Non-Hispanic Multiple Race	Not Hispanic or Latino (38003564)/Multiple Race (8522)	EHR/Person

Middle Eastern and North African (MENA)	Not Hispanic or Latino (38003564)/MENA (38003615)	EHR/Person
Non-Hispanic Native Hawaiian or Other Pacific Islander	Not Hispanic or Latino (38003564)/Native Hawaiian or Other Pacific Islander (8557)	EHR/Person
Non-Hispanic White	Not Hispanic or Latino (38003564)/White (8527)	EHR/Person
**Number of previous breast biopsies**		
Biopsy, breast, with placement of breast localization device(s) and imaging of the biopsy specimen, percutaneous; first lesion, including ultrasound guidance	44816376	EHR/Procedure
Biopsy, breast, with placement of breast localization device(s) and imaging of the biopsy specimen, percutaneous; first lesion, including stereotactic guidance	44816374	EHR/Procedure
Image guided placement, metallic localization clip, percutaneous, during breast biopsy/aspiration	2102715	EHR/Procedure
Biopsy of breast; percutaneous, automated vacuum assisted or rotating biopsy device, using imaging guidance	2102675	EHR/Procedure
Biopsy of breast; percutaneous, needle core, using imaging guidance	2102674	EHR/Procedure
Stereotactic localization guidance for breast biopsy/needle placement, each lesion, radiological supervision and interpretation	2211806	EHR/Procedure
Biopsy of breast	4047494	EHR/Procedure
Biopsy, breast, with placement of breast localization device(s) and imaging of the biopsy specimen, percutaneous; first lesion, including magnetic resonance guidance	44816378	EHR/Procedure
Biopsy, breast, with placement of breast localization device(s) and	44816377	EHR/Procedure

imaging of the biopsy specimen, percutaneous; each additional lesion, including ultrasound guidance		
Percutaneous needle biopsy of breast	4028790	EHR/Procedure
Stereotactic localization guidance for breast biopsy/needle placement (Deprecated)	42737563	EHR/Procedure
Biopsy of breast; percutaneous, needle core, not using imaging guidance	2102672	EHR/Procedure
Biopsy, breast, with placement of breast localization device(s) and imaging of the biopsy specimen, percutaneous; each additional lesion, including stereotactic guidance	44816375	EHR/Procedure
Biopsy of breast; open, incisional	2102673	EHR/Procedure
Open biopsy of breast	42539496	EHR/Procedure
Biopsy, breast, with placement of breast localization device(s) and imaging of the biopsy specimen, percutaneous; each additional lesion, including magnetic resonance guidance	44816379	EHR/Procedure
Vacuum assisted biopsy of breast using ultrasound	4331230	EHR/Procedure
Core needle biopsy of breast using ultrasound	4196880	EHR/Procedure
Excisional biopsy of breast	4028789	EHR/Procedure
Excisional biopsy of breast mass	4172166	EHR/Procedure
Breast biopsy and related procedures	4129191	EHR/Procedure
Core needle biopsy of breast	4195312	EHR/Procedure
**Mammographic History**		

Indicator for abnormal mammogram condition	4059049	EHR/Condition
Indicator for microcalcification in the breast	45757639	EHR/Condition
Mammography procedure (general)	4324693	EHR/Procedure
Mammogram specific to the left breast	37109442	EHR/Procedure
Mammography for both breasts	4182722	EHR/Procedure
Diagnostic mammography procedure	40218694	EHR/Procedure
Diagnostic breast tomosynthesis procedure	45890623	EHR/Procedure
Date when the last mammogram was performed	4324905	EHR/Observation
Screening measurement for both breasts	36713187	EHR/Lab/Measurements
**Number of First-degree relatives with breast cancer**	Question: 836772, Answer: Mother (43528504), Sibling (43528505), Daughter (43528501), Father (43528502), and Son (43528506). All other responses did not meet predictor inclusion criteria.	Survey/Personal and Family Health History
**Outcome: EHR Breast Cancer Diagnosis**		
**Primary malignant neoplasm of female breast (parent code)**	137809	EHR/Condition
Primary malignant neoplasm of upper outer quadrant of female breast	436353	EHR/Condition
Overlapping malignant neoplasm of female breast	133711	EHR/Condition
Primary malignant neoplasm of central portion of female breast	432845	EHR/Condition
Primary malignant neoplasm of upper inner quadrant of female breast	440956	EHR/Condition
Primary malignant neoplasm of lower outer quadrant of female breast	441515	EHR/Condition
Primary malignant neoplasm of lower inner quadrant of female breast	432263	EHR/Condition

Primary malignant neoplasm of female left breast	36684818	EHR/Condition
Primary malignant neoplasm of axillary tail of left female breast	36684817	EHR/Condition
Infiltrating duct carcinoma of left female breast	759932	EHR/Condition
Infiltrating lobular carcinoma of left female breast	36712724	EHR/Condition
Primary malignant neoplasm of female right breast	765123	EHR/Condition
Primary malignant neoplasm of axillary tail of right female breast	36684820	EHR/Condition
Infiltrating duct carcinoma of right female breast	759933	EHR/Conditions
Infiltrating ductal carcinoma of upper outer quadrant of right female breast	36712723	EHR/Condition
Infiltrating lobular carcinoma of right female breast	36712725	EHR/Condition
Infiltrating duct carcinoma of female breast	40492507	EHR/Condition
Primary malignant neoplasm of areola of female breast	433148	EHR/Condition
**Outcome: Survey Breast Cancer Self-Report**		
Including yourself, who in your family has had breast cancer? Select all that apply	Question: 836772, Answer: Self (43528500). All other responses did not meet outcome inclusion criteria.	Survey/Personal and Family Health History
Are you still seeing a doctor or health care provider for breast cancer?	Question: 43530286, Answer: Yes (43529900). All other responses did not meet outcome inclusion criteria.	Survey/Personal and Family Health History
About how old were you when you were first told you had cancer?	Question: 43530444, Answer: Child (0-11) (1384758), Adolescent (12-17) (1384903), Adult (18-64) (1384871), Older adult (65-74) (1385393), and Elderly (75+) (1385308). All other responses did not meet outcome inclusion criteria.	Survey/Personal and Family Health History
Are you currently prescribed medications and/or receiving treatment for breast cancer?	Question: 4358770, Answer: Yes (4188539). All other responses did not meet outcome inclusion criteria.	Survey/Personal and Family Health History

[a]Age at consent, participants date of birth minus the AoU primary consent date.

[b]Concept Id, Observational Medical Outcomes Partnership (OMOP) Codes for EHR Id's and Question/Answer Codes for Survey Id's.

Supplemental Table 2. American Community Survey (ACS) 2017 Measures for Area Deprivation Index Calculation[a].

Measure	Description	ACS Variable Codes	ACS Code Title
Zip code	3-digit zip code		
Fraction assisted income	fraction of households receiving public assistance income or food stamps or SNAP in the past 12 months	B19058 (001, 002)	Public Assistance Income or Food Stamps/SNAP for Households
Fraction high school education	fraction of population 25 and older with educational attainment of at least high school graduation (includes GED equivalency)	B15003 (001, 017: 025)	Educational Attainment
Median household income	median household income in the past 12 months in 2015 inflation-adjusted dollars	B19013 (001)	Median Household Income
Fraction no health insurance	fraction of population with no health insurance coverage	B27010 (001, 017, 033, 050, 066)	Types of Health Insurance Coverage by Age
Fraction poverty	fraction of population with income in past 12 months below poverty level	B17001 (001, 002)	Poverty Status by Sex by Age
Fraction vacant housing	fraction of houses that are vacant	B25002 (001, 003)	Occupancy Status
Area deprivation index	a deprivation index for each census tract in the United States based on a principal components analysis of six different 2017ACS measures; rescaling and normalizing forces the index to range from 0 to 1, with a higher index being more deprived.		

[a]Area deprivation index calculation steps: 1. zip-codes formatted to Federal Information Processing Standards (FIPS) codes, 2. population weight = census tract population*residential ratio, excluding zero weights, and 3. weighted average of six socioeconomic measures (1. fraction assisted income, 2. fraction high school education, 3. median household income, 4. fraction no health insurance, 5. fraction poverty, 6. fraction vacant housing).

Supplemental Table 3. Neighborhood-Based Indicators.

Survey Question	Answer Option	Subscale	Scoring
**Social Cohesion Scale**			
How much do you agree that people around here are willing to help their neighbor?	Strongly agree = 5, Agree = 4, Neutral (neither agree nor disagree) = 3, Disagree = 2, Strongly disagree = 1, Did not answer = NA		Sum recorded score. A higher composite score equates to stronger connections with neighbors, more trust, more social interactions, and shared values.
How much do you agree that people in your neighborhood generally get along with each other?	Strongly agree = 5, Agree = 4, Neutral (neither agree nor disagree) = 3, Disagree = 2, Strongly disagree = 1, Did not answer = NA		
How much do you agree that people in your neighborhood can be trusted?	Strongly agree = 5, Agree = 4, Neutral (neither agree nor disagree) = 3, Disagree = 2, Strongly disagree = 1, Did not answer = NA		

How much do you agree that people in your neighborhood share the same values?	Strongly agree = 5, Agree = 4, Neutral (neither agree nor disagree) = 3, Disagree = 2, Strongly disagree = 1, Did not answer = NA		
**Ross-Mirowsky Disorder Scale**			
How much you agree or disagree that there is a lot of graffiti in your neighborhood?	Strongly agree = 4, Agree = 3, Disagree = 2, Strongly disagree = 1	Physical Disorder	Sum recorded score. A higher composite score equates to higher levels of distress.
How much you agree or disagree that your neighborhood is noisy?	Strongly agree = 4, Agree = 3, Disagree = 2, Strongly disagree = 1	Physical Disorder	
How much you agree or disagree that vandalism is common in your neighborhood?	Strongly agree = 4, Agree = 3, Disagree = 2, Strongly disagree = 1	Physical Disorder	
How much you agree or disagree that there are lot of abandoned buildings in your neighborhood?	Strongly agree = 4, Agree = 3, Disagree = 2, Strongly disagree = 1	Physical Disorder	
How much you agree or disagree that there are too many people hanging around on the streets near your home?	Strongly agree = 4, Agree = 3, Disagree = 2, Strongly disagree = 1	Social Disorder	
How much you agree or disagree that there is a lot of crime in your neighborhood?	Strongly agree = 4, Agree = 3, Disagree = 2, Strongly disagree = 1	Social Disorder	
How much you agree or disagree that there is too much drug use in your neighborhood?	Strongly agree = 4, Agree = 3, Disagree = 2, Strongly disagree = 1	Social Disorder	
How much you agree or disagree that there is too much alcohol use in your neighborhood?	Strongly agree = 4, Agree = 3, Disagree = 2, Strongly disagree = 1	Social Disorder	
How much you agree or disagree that you are always having trouble with your neighbors?	Strongly agree = 4, Agree = 3, Disagree = 2, Strongly disagree = 1	Social Disorder	
How much you agree or disagree that your neighborhood is clean?	Reverse Scoring: Strongly agree = 1, Agree = 2, Disagree = 3, Strongly disagree = 4	Physical Order	
How much you agree or disagree that people in your neighborhood take good care of their houses and apartments?	Reverse Scoring: Strongly agree = 1, Agree = 2, Disagree = 3, Strongly disagree = 4	Physical Order	
How much you agree or disagree that in your neighborhood people watch out for each other?	Reverse Scoring: Strongly agree = 1, Agree = 2, Disagree = 3, Strongly disagree = 4	Social Order	
How much you agree or disagree that your neighborhood is safe?	Reverse Scoring: Strongly agree = 1, Agree = 2, Disagree = 3, Strongly disagree = 4	Social Order	
**Physical Activity Neighborhood Environment Scale**			
What is the main type of housing in your neighborhood?	Detached single-family housing = 0, Townhouses, row house, apartments, or condos of 2–3 stories = 1, Mix of single-family residences and townhouses/apartments/condos = 1, Apartments or condos of 4–12 stories = 1, Apartments or condos of more than 12 stories = 1		Sum recorded scores. A higher composite score equates to a more supportive perceived

Many shops, stores, markets or other places to buy things I need are within easy walking distance of my home.	Strongly agree = 4, Agree = 3, Disagree = 2, Strongly disagree = 1, NA = Missing		neighborhood for physical activity.
It is within a 10–15 minute walk to a transit stop (bus, train, trolley, or tram) from my home.	Strongly agree = 4, Agree = 3, Disagree = 2, Strongly disagree = 1, NA = Missing		
There are sidewalks on most of the streets in my neighborhood.	Strongly agree = 4, Agree = 3, Disagree = 2, Strongly disagree = 1, NA = Missing		
There are facilities to bicycle in or near my neighborhood (e.g., special lanes, separate paths, or shared use paths for cycles and pedestrians).	Strongly agree = 4, Agree = 3, Disagree = 2, Strongly disagree = 1, NA = Missing		
My neighborhood has several free or low-cost recreation facilities (such as parks, walking trails, bike paths, recreation centers, playgrounds, public swimming pools).	Strongly agree = 4, Agree = 3, Disagree = 2, Strongly disagree = 1, NA = Missing		
The crime rate in my neighborhood makes it unsafe to go on walks at night.	Strongly agree = 1, Agree = 2, Disagree = 3, Strongly disagree = 4, NA = Missing		
The crime rate in my neighborhood makes it unsafe to go on walks during the day.	Strongly agree = 1, Agree = 2, Disagree = 3, Strongly disagree = 4, NA = Missing		

Supplemental Table 4. DeNovo Model Fit Across Analytic Subgroups.

	Metric	Model 0[a] (BCRM)	Model 1[b]	Model 2[c] (NBRM)
Prospective	Mean AIC	6577·2	6565·3	6586·7
Ret-G1		2807·7	2789·7	2806·9
Ret-G2		3607·3	3616·5	3612·2
Ret-G3		3200·1	3186·3	3179·9
Ret-G4		1961·7	1790·4	1784·7
Survey-Only		6087·7	6070·1	6244·3
Prospective	Mean BIC	6652·7	6716·3	6707·7
Ret-G1		2872·7	2918·4	2910·9
Ret-G2		3674·9	3751·8	3721·5
Ret-G3		3266·4	3318·9	3287·0
Ret-G4		5039·7	3944·5	3956·4
Survey-Only		6163·3	6221·3	6366·5
Prospective	Mean McFadden Pseudo-R	0·04	0·05	0·04
Ret-G1		0·04	0·05	0·04
Ret-G2		0·04	0·04	0·04
Ret-G3		0·05	0·07	0·06
Ret-G4		0·06	0·15	0·15
Survey-Only		0·12	0·12	0·09

Abbreviations: AIC, Akaike Information Criterion; BIC, Bayesian Information Criterion; BCRM, Breast Cancer Risk Model (clinical factors, race, ethnicity); NBRM, Neighborhood-Based Risk Model (clinical factors, neighborhood indicators); T1, age at consent; nbiop, number of biopsies; nrels, number of first-degree relatives with cancer; social scale, Social Cohesion Scale; rm scale, Ross-Mirowsky Disorder Scale; panes scale, Physical Activity Neighborhood Environment Scale; ADI, area deprivation index.

[a]Model 0 (BCRM): breastcancer ~ T1 + nbiop + nrels + race and ethnicity.
[b]Model 1 (BCRM + neighborhood indicators): breastcancer ~ T1 + nbiop + nrels + race and ethnicity + social scale + rm scale + panes scale + ADI.
[c]Model 2 (NBRM): breastcancer ~ T1 + nbiop + nrels + social scale + rm scale + panes scale + ADI.

Supplemental Table 5. Neighborhood-Based Indicator Variance Explained by Race and Ethnicity.

	Race and Ethnicity
**Neighborhood-Based Indicators**	**Eta-squared (η2)**
Social Cohesion Scale	0·03
Ross-Mirowsky Disorder Scale	0·04
Physical Activity Neighborhood Environment Scale	0·001
Area Deprivation Index	0·12

Supplemental Table 6. Analytic Subgroup Population by Invasive and Invasive + DCIS Datasets.

	Prospective		Ret-G1		Ret-G2		Ret-G3		Ret-G4		Survey-only	
	Case	Control	Case	Control	Case	Control	Case	Control	Case	Control	Case	Control
**Total Invasive**	3,081	9,231	1,309	3,918	1,679	5,036	1,516	4,541	934	2,794	3,098	9,290
**Total Invasive + DCIS**	3,313	9,924	1,441	4,314	1,700	5,098	1,521	4,561	934	2,797	2,922	8,760
**Difference**	232	693	132	396	21	62	5	20	0	3	-176	-530

Abbreviations: DCIS, Ductal carcinoma in situ; Prospective, prospective diagnoses after All of Us (AoU) consent with matched controls; Ret-G1, retrospective diagnoses 0-2 years before All of Us consent with matched controls; Ret-G2, retrospective diagnoses 2-5 years before AoU consent with matched controls; Ret-G3, retrospective diagnoses 5-10 years before AoU consent with matched controls; Ret-G4, retrospective diagnoses 10+ years before AoU consent with matched controls; and Survey-only, participants with a breast cancer diagnosis determined only by AOU survey with no diagnosis date available.

Supplemental Table 7. Analytic Subgroup Discrimination Comparison Between Invasive and Invasive + DCIS Datasets Across Models by Race and Ethnicity.

	Pooled AUC											
	Prospective		Ret-G1		Ret-G2		Ret-G3		Ret-G4		Survey-only	
**Model 0[a]**	**Imputed Invasive**	**Imputed Invasive + DCIS**	**Imputed Invasive**	**Imputed Invasive + DCIS**	**Imputed Invasive**	**Imputed Invasive + DCIS**	**Imputed Invasive**	**Imputed Invasive + DCIS**	**Imputed Invasive**	**Imputed Invasive + DCIS**	**Imputed Invasive**	**Imputed Invasive + DCIS**
Overall	0.55	0.54	0.53	0.56	0.53	0.54	0.54	0.53	0.54	0.55	0.61	0.61
Non-Hispanic Black	0.57	0.59	0.59	0.55	0.54	0.50	0.58	0.56	0.48	0.63	0.60	0.65
Hispanic	0.51	0.51	0.51	0.43	0.50	0.50	0.41	0.47	0.50	0.55	0.64	0.62

Non-Hispanic White	0.51	0.52	0.54	0.54	0.53	0.55	0.54	0.54	0.54	0.55	0.55	0.54
**Model 1[b]**												
Overall	0.56	0.54	0.54	0.57	0.54	0.54	0.55	0.54	0.61	0.63	0.61	0.61
Non-Hispanic Black	0.56	0.58	0.59	0.60	0.54	0.50	0.57	0.55	0.53	0.71	0.61	0.60
Hispanic	0.54	0.52	0.51	0.44	0.51	0.52	0.44	0.49	0.59	0.67	0.64	0.59
Non-Hispanic White	0.52	0.52	0.54	0.56	0.53	0.55	0.55	0.55	0.60	0.63	0.55	0.54
**Model 2[c]**												
Overall	0.55	0.54	0.53	0.56	0.54	0.54	0.55	0.54	0.61	0.62	0.59	0.58
Non-Hispanic Black	0.57	0.59	0.59	0.60	0.54	0.50	0.57	0.55	0.53	0.71	0.62	0.60
Hispanic	0.53	0.52	0.50	0.44	0.51	0.52	0.44	0.49	0.59	0.66	0.65	0.59
Non-Hispanic White	0.52	0.53	0.54	0.56	0.53	0.55	0.55	0.55	0.60	0.62	0.55	0.54

Abbreviations: DCIS, Ductal carcinoma in situ; AUC, Area under the curve; BCRM, Breast Cancer Risk Model (clinical factors, race, ethnicity); NBRM, Neighborhood-Based Risk Model (clinical factors, neighborhood indicators); T1, age at consent; nbiop, number of previous biopsies; nrels, number of first-degree relatives with breast cancer; social scale, Social Cohesion scale; rm scale, Ross-Mirowsky Disorder scale; panes scale, Physical Activity Neighborhood Environment (PANES) scale; ADI, area deprivation index; Prospective, prospective diagnoses after All of Us (AoU) consent with matched controls; Ret-G1, retrospective diagnoses 0-2 years before All of Us consent with matched controls; Ret-G2, retrospective diagnoses 2-5 years before AoU consent with matched controls; Ret-G3, retrospective diagnoses 5-10 years before AoU consent with matched controls; Ret-G4, retrospective diagnoses 10+ years before AoU consent with matched controls; and Survey-only, participants with a breast cancer diagnosis determined only by AOU survey with no diagnosis date available.

[a]Model 0 (BCRM): breastcancer ~ T1 + nbiop + nrels + race and ethnicity.
[b]Model 1 (BCRM + neighborhood indicators): breastcancer ~ T1 + nbiop + nrels + race and ethnicity + social scale + rm scale + panes scale + ADI.
[c]Model 2 (NBRM): breastcancer ~ T1 + nbiop + nrels + social scale + rm scale + panes scale + ADI.

Ethnoracial categories, Non-Hispanic Asian, Non-Hispanic Native Hawaiian or Pacific Islander, and Other, not shown due to small participant counts and unstable estimates, but are included in overall pooled AUC.

Supplemental Table 8. Analytic Subgroup Discrimination Comparison Between Imputed and Complete Case Datasets Across Models by Race and Ethnicity.

	Prospective		Ret-G1		Ret-G2		Ret-G3		Ret-G4		Survey-only	
	**Imputed**	**Complete case**	**Imputed**	**Complete case**	**Imputed**	**Complete case**	**Imputed**	**Complete case**	**Imputed**	**Complete case**	**Imputed**	**Complete case**
**Model 0[a]**	**Pooled AUC**	**AUC**	**Pooled AUC**	**AUC**	**Pooled AUC**	**AUC**	**Pooled AUC**	**AUC**	**Pooled AUC**	**AUC**	**Pooled AUC**	**AUC**
Overall	0.55	0.52	0.53	0.55	0.53	0.54	0.54	0.54	0.54	0.56	0.61	0.54
Non-Hispanic Black	0.57	0.61	0.59	0.53	0.54	0.45	0.58	0.56	0.48	0.58	0.60	0.51
Hispanic	0.51	0.53	0.51	0.54	0.50	0.52	0.41	0.37	0.50	0.44	0.64	0.60
Non-Hispanic White	0.51	0.51	0.54	0.56	0.53	0.56	0.54	0.54	0.54	0.57	0.55	0.53
**Model 1[b]**												
Overall	0.56	0.54	0.54	0.56	0.54	0.55	0.55	0.54	0.61	0.62	0.61	0.55
Non-Hispanic Black	0.56	0.61	0.59	0.55	0.54	0.50	0.57	0.56	0.53	0.65	0.61	0.53
Hispanic	0.54	0.56	0.51	0.55	0.51	0.53	0.44	0.39	0.59	0.35	0.64	0.62
Non-Hispanic White	0.52	0.52	0.54	0.58	0.53	0.57	0.55	0.55	0.60	0.63	0.55	0.55
**Model 2[c]**												
Overall	0.55	0.53	0.53	0.56	0.54	0.55	0.55	0.54	0.61	0.62	0.59	0.55
Non-Hispanic Black	0.57	0.61	0.59	0.54	0.54	0.50	0.57	0.57	0.53	0.65	0.62	0.53
Hispanic	0.53	0.57	0.50	0.54	0.51	0.54	0.44	0.39	0.59	0.38	0.65	0.61
Non-Hispanic White	0.52	0.52	0.54	0.58	0.53	0.57	0.55	0.55	0.60	0.63	0.55	0.55

Abbreviations: DCIS, Ductal carcinoma in situ; AUC, Area under the curve; BCRM, Breast Cancer Risk Model (clinical factors, race, ethnicity); NBRM, Neighborhood-Based Risk Model (clinical factors, neighborhood indicators); T1, age at consent; nbiop, number of previous biopsies; nrels, number of first-degree relatives with breast cancer; social scale, Social Cohesion scale; rm scale, Ross-Mirowsky Disorder scale; panes scale, Physical Activity Neighborhood Environment (PANES) scale; ADI, area deprivation index; Prospective, prospective diagnoses after All of Us consent with matched controls; Ret-G1,

retrospective diagnoses 0-2 years before All of Us (AoU) consent with matched controls; Ret-G2, retrospective diagnoses 2-5 years before AoU consent with matched controls; Ret-G3, retrospective diagnoses 5-10 years before AoU consent with matched controls; Ret-G4, retrospective diagnoses 10+ years before AoU consent with matched controls; and Survey-only, participants with a breast cancer diagnosis determined only by AOU survey with no diagnosis date available.

[a]Model 0 (BCRM): breastcancer ~ T1 + nbiop + nrels + race and ethnicity.
[b]Model 1 (BCRM + neighborhood indicators): breastcancer ~ T1 + nbiop + nrels + race and ethnicity + social scale + rm scale + panes scale + ADI.
[c]Model 2 (NBRM): breastcancer ~ T1 + nbiop + nrels + social scale + rm scale + panes scale + ADI.

Ethnoracial categories, Non-Hispanic Asian, Non-Hispanic Native Hawaiian or Pacific Islander, and Other, not shown due to small participant counts and unstable estimates, but are included in overall pooled AUC.
